# Thromboelastography in Patients with Inflammatory Bowel Disease

**DOI:** 10.1155/2020/3245657

**Published:** 2020-05-20

**Authors:** Yonghua Shen, Liangliang Shi, Juanjuan Zhang, Hao Zhu, Yuling Yao, Zhenqing Liu, Xiaoping Zou, Xiaoqi Zhang

**Affiliations:** ^1^Department of Gastroenterology, The Affiliated Drum Tower Hospital of Nanjing University, Medical School, No. 321, Zhongshan Road, Nanjing 210008, China; ^2^Department of Reproduction, Affiliated Nanjing Maternal and Child Health Hospital, Nanjing Medical University, Nanjing 210004, China; ^3^Division of Stem Cell Biology Research, Department of Developmental and Stem Cell Biology, Beckman Research Institute of City of Hope, Duarte, CA 91010, USA

## Abstract

**Purpose:**

Patients with inflammatory bowel disease (IBD) frequently suffer from venous thromboembolic events, and the risk of thromboembolism increases along with disease activity. This study was conducted to discover novel thrombophilic markers using thromboelastography (TEG) and to evaluate the relation between the predisposing factors and the activity of disease in Chinese patients with Crohn's disease (CD) and ulcerative colitis (UC).

**Methods:**

Thirty-four patients with CD, 29 patients with UC, and 53 healthy volunteers were enrolled into this study. Blood levels of *R*, *K*, *α* Angle, *G*, maximal amplitude (MA), and LY30 with TEG were determined.

**Results:**

Mean values of *R*, *K*, *α* Angle, *G*, and MA were significantly different in patients with CD and UC compared with the healthy individuals. Patients with active CD had different *K*, *α* Angle, *G*, and MA levels compared with patients in remission (*P* < 0.05, *P* < 0.001, *P* < 0.001, and *P* < 0.001). Levels of *R*, *α* Angle, *G*, and MA were also significantly different in active UC patients compared with those in remission (*P* < 0.01, *P* < 0.001, *P* < 0.001, and *P* < 0.001). Except for the *G* level in the CD group, differences in all TEG levels between healthy individuals and IBD patients in remission were not statistically significant. No statistical differences were observed in LY30 among patients with active phase, patients in remission, and the healthy individuals.

**Conclusion:**

Thrombophilic defects are common in Chinese patients with IBD, and TEG can be considered a new direction to anticoagulant thromboprophylaxis in IBD.

## 1. Introduction

Inflammatory bowel disease (IBD), including Crohn's disease (CD) and ulcerative colitis (UC), is a chronic and relapsing inflammatory systemic disease which primarily affects the bowels and causes extraintestinal manifestations simultaneously [1, 2]. The patients with IBD have an approximately 3-fold higher risk of venous thromboembolism (VTE) compared with persons without IBD, and the risk of VTE increases along with disease activity [3, 4]. Anticoagulant thromboprophylaxis is recommended in active IBD given that there is no severe active bleeding [5]. Several studies using various markers of the coagulation system have revealed the risk of thrombosis in IBD patients, such as protein C, protein S, and antithrombin III [6, 7]. However, the heterogeneity in the identified studies remains and the precise mechanism of hypercoagulability in IBD is not understood well.

Thromboelastography (TEG) measures the integrated dynamics of the coagulation process from clot formation to clot consistency, which provides global information about the balance between both sides of coagulation, clot strength, and lysis [8]. Therefore, TEG is likely to be valuable for appraising coagulation status and the response to anticlotting therapy. Currently, TEG has been used in various clinical conditions, such as in pregnant patients, in obstetric patients, liver transplantation, cardiac surgery, myocardial infarction, and in trauma patients [9–14]. One of the most crucial advantages of TEG is the comprehensive global assessment to the coagulation process preceding routine tests [15]. However, TEG was not reported in IBD with an abnormal coagulation status till now.

The present study was aimed at determining whether TEG was valuable in assessing hypercoagulable states of IBD. Our findings will provide novel references to anticoagulant thromboprophylaxis in IBD.

## 2. Methods

### 2.1. Patients and Healthy Volunteers

This study was conducted with the approval of the local research ethics committee. Written consent for this study was obtained from each subject before enrollment. Sixty-three patients with IBD followed up at the Department of Gastroenterology of the Affiliated Drum Tower Hospital of Medical School of Nanjing University were enrolled in this study. In the CD group, there were 22 men and 12 women, with age ranging from 16 to 53 yr. The UC group included 13 men and 16 women, with age ranging from 19 to 54 yr. Fifty-three healthy volunteers were enrolled as the control group. There were no statistical differences for gender and age among the three groups. The diagnosis of CD and UC was on the basis of standard criteria [16]. Crohn's Disease Activity Index (CDAI) score was used to evaluate disease activity in CD [17], and the modification of Mayo Score system was used in UC [18, 19]. A CDAI score equal or higher than 150 in patients with CD and a Mayo score equal or higher than 3 in patients with UC were considered to manifest clinical active disease. None of the IBD patients and healthy volunteers had a history of previous thromboembolism. The clinical data of IBD patients and controls are summarized in Table 1.

### 2.2. Laboratory Studies

Whole blood was collected by expert nurses with a clean venipuncture at the fossa cubitalis. The first 5 ml of each sample was discarded. 5-8 ml of whole blood was then collected into a 10 ml citrated tube. The samples were kept at 15-25°C and analyzed within 2 hours from sampling. Standard TEG was performed with the TEG 5000 Thrombelastograph Hemostasis Analyzer System (Haemonetics Corporation, Niles, USA) according to the manufacturer's protocol. The following values were evaluated with a TEG test: *R* (minutes), *K* (minutes), *α* Angle (degrees), *G* (dynes/cm2), maximal amplitude (MA, mm), and LY30 (percentage). *R* is the time until the TEG tracing amplitude returns to 2 mm, and *K* is derived from *R* until the amplitude reaches 20 mm which reflects the speed of clot strengthening. *α* Angle is generated by the slope of TEG tracing from the horizontal line of *R* and also represents clot strengthening. MA measures the maximal strength of the clot, and *G* reflects the clot strength or firmness. LY30, recorded as percent lysis, measures the clot lysis as the decay with MA over 30 minutes.

The TEG instrument was validated for quality assurance through daily quality control procedures with normal and abnormal controls for operational checks and calibration verification. The quality control methods are based on the recommendations of the Clinical and Laboratory Standards Institute, the US Clinical Laboratory Improvement Amendments, and the performance standards of TEG analyzer.

### 2.3. Statistical Analysis

The data were expressed as mean ± SD and were analyzed with SPSS software, version 19.0 (SPSS Inc., Chicago, IL, USA). Statistical differences were evaluated by one-way ANOVA, using LSD, SNK and Dunnett's methods. Results were considered statistically significant differences when the analysis reached a *P* value of < 0.05.

## 3. Results

The distribution of CD showed 55.9% ileocolic, 26.5% colonic, and 17.6% ileal involvement. UC extent was 58.6% pancolitis, 34.5% left-sided, and 6.9% proctitis. There were 7 abscesses, 3 intestinal strictures, 6 perianal fistulas, and 1 alimentary tract bleeding in CD patients. Only one complication of toxic megacolon appeared in UC patients. Twenty-one (61.8%) patients with CD and 19 (65.5%) patients with UC showed active disease (Table 1).

*R* and *K* levels were statistically lower in patients with CD compared with healthy individuals (*P* < 0.01, *P* < 0.05). *α* Angle, *G*, and MA were significantly higher in patients with CD compared with healthy individuals (*P* < 0.001, *P* < 0.001, and *P* < 0.001). Mean values of *R* and *K* were statistically lower in patients with UC than in the healthy control group (*P* < 0.001, *P* < 0.05). Mean values of *α* Angle, *G*, and MA were significantly higher in patients with UC than in the healthy control group (*P* < 0.001, *P* < 0.001, and *P* < 0.001). There was no difference between IBD patients and healthy individuals in LY30 (Table 2).

Further, levels of TEG in patients with active phase were compared with the patients in remission. Mean values of *R* were statistically lower in patients with active phase compared with patients in remission (*P* < 0.01). Levels of *α* Angle, *G*, and MA were significantly higher in patients with active phase than patients in remission (*P* < 0.001, *P* < 0.001, and *P* < 0.001). There were statistical differences in *R* and *G* between IBD patients in remission and healthy individuals (*P* < 0.05, *P* < 0.05) (Table 3).

Patients with CD and UC were also evaluated separately. Patients with active CD had lower *K* and higher *α* Angle, *G*, and MA levels than patients in remission (*P* < 0.05, *P* < 0.001, *P* < 0.001, and *P* < 0.001). Patients with active UC had lower *R* and higher *α* Angle, *G*, and MA levels than patients in remission (*P* < 0.01, *P* < 0.001, *P* < 0.001, and *P* < 0.001). Except for the *G* level in the CD group (*P* < 0.05), differences in all TEG levels between healthy individuals and patients in remission in both CD and UC groups were not statistically significant. No statistical differences were observed in LY30 among patients with active phase, patients in remission, and the healthy individuals (Table 3).

## 4. Discussion

Recently, views on the hypercoagulability of patients with IBD have made a major change. This study is the first to appraise the value of TEG in IBD patients. Our primary finding was that IBD patients were more hypercoagulable with various TEG parameters compared to controls. This was evidenced by statistical differences in *R*, *K*, *α* Angle, *G*, and MA, but no differences in LY30. Another important finding was that active IBD patients were hypercoagulable on some TEG parameters compared to patients in remission. We cannot deduce if the abnormalities reflected on TEG predated the hypercoagulability of IBD patients or were the consequences thereof. However, we indeed found substantial variability in the coagulation process within IBD patients as reflected in TEG values. *R* reflects coagulation factor activity, which was the most affected parameter in all analyzed TEG variables by specific coagulation factor deficiencies [20]. *K* and *α* Angle are supposed to represent the propagation phase of the enzymatic factors related to clot strengthening, which is mostly accomplished by fibrin polymerization and fibrinogen cleavage in the phase of clotting [21]. *G* values reveal the strength of mature clot. Most of the total *G* is attributed to the interaction between the platelet and fibrin [22]. MA represents the greatest strength accomplished by the clot, which assesses the combination of platelet quantity and function as well as the activity of fibrinogen rather than a simple effect [23]. LY30 is calculated 30 minutes after achieving MA with the percent reduction of clot strength as a standard measurement of fibrinolysis [24]. Of all the detective TEG indicators, only LY30 has no significant difference from the control group in CD and UC patients, which means that the fibrinolysis function of IBD patients is not impaired and the mechanisms of hypercoagulable state can be suggested in IBD. Interestingly, some TEG indicators were statistically different between activity and the remission period in IBD, but some were not in this study. Such discrepancy may be related to the subjectivity of CD and UC staging.

At present, the data were sufficient to determine TEG as a method that might be valuable in assisting us to understand the target of anticoagulant thromboprophylaxis therapy better. However, the study still had several limitations. First, this study could be defective because of the various operators which carried out TEG detection. To minimize the deviation of TEG based on the existing imprecision, daily quality control was carried out carefully. In addition, studies in large population are needed to reduce imprecision with TEG detection as far as possible. Second, during the treatment of IBD, drugs such as glucocorticoids and immunosuppressants may affect the TEG results and become a confounding variable. Finally, we only measured the values of TEG, while the molecular mechanisms of coagulation and fibrinolysis were not be explored in depth. However, we think relevant molecular markers will gradually be explored with the subsequent studies.

## 5. Conclusions

This study demonstrates that hypercoagulability exists in many IBD patients and is related to disease activity. Further study on the basis of present data will determine if TEG measuring is valuable for the dynamic aspects of coagulation and anticoagulant thromboprophylaxis therapy.

## Figures and Tables

**Table 1:** Clinical characteristics of the patients with IBD and the controls in the study.

	Control group (*n* = 53)	CD (*n* = 34)	UC (*n* = 29)
Age (years)	34.7 ± 10.4	34.9 ± 10.9	35.8 ± 11.6
Sex			
Male	27 (50.9%)	22 (64.7%)	13 (44.8%)
Female	26 (49.1%)	12 (35.3%)	16 (55.2%)
Duration of disease (months)	—	99.3 ± 56.2	167.0 ± 130.0
Localization of disease	—	Ileitis 6 (17.6%)	Proctitis 2 (6.9%)
		Colitis 9 (26.5%)	Left-sided 10 (34.5%)
		Ileocolitis 19 (55.9%)	Pancolitis 17 (58.6%)
Complications	—	Abscess 7	Toxic megacolon 1
		Intestinal stricture 3	
		Perianal fistula 6	
		Alimentary tract bleeding 1	
Clinical activity			
Active	—	21 (61.8%)	19 (65.5%)
Inactive		13 (38.2%)	10 (34.5%)

IBD: inflammatory bowel disease; CD: Crohn's disease; UC: ulcerative colitis.

**Table 2:** Comparison of thrombophilic markers in TEG between the IBD and control groups.

	Control group (*n* = 53)	CD (*n* = 34)	UC (*n* = 29)
*R* (min)	6.09 ± 1.26	5.78 ± 0.84^a,2^	5.39 ± 1.09^a,3^
*K* (min)	1.74 ± 0.56	1.48 ± 0.57^a,1^	1.53 ± 0.42^a,1^
*α* Angle (°)	65.82 ± 6.92	69.57 ± 7.76^a,3^	67.77 ± 6.11^a,3^
G (dynes/cm2)	8352.74 ± 1409.24	10966.15 ± 3493.87^a,3^	9250.83 ± 2173.02^a,3^
MA (mm)	58.11 ± 4.91	62.51 ± 8.83^a,3^	60.59 ± 7.01^a,3^
LY30 (%)	2.17 ± 1.25	2.30 ± 1.04	1.74 ± 1.16

CD: Crohn's disease; UC: ulcerative colitis. ^a^Compared with the control group. ^1^*P* < 0.05, ^2^*P* < 0.01, and ^3^*P* < 0.001.

**Table 3:** Comparative analysis of TEG markers in active IBD patients versus those patients in remission and control group.

	*R* (min)	*K* (min)	*α* Angle (°)	*G* (dynes/cm^2^)	MA (mm)	LY30 (%)
Control group (*n* = 53)	6.09 ± 1.26	1.74 ± 0.56	65.82 ± 6.92	8352.74 ± 1409.24	58.11 ± 4.91	2.17 ± 1.25
IBD						
Remission (*n* = 23)	5.79 ± 0.81^a1^	1.61 ± 0.57	65.69 ± 7.96	8341.23 ± 2717.16^a1^	58.41 ± 7.61	2.06 ± 1.04
Activity (n = 40)	5.49 ± 1.04^a3b2^	1.44 ± 0.44^a2^	69.87 ± 6.67^a3b3^	11010.06 ± 2919.47^a3,b3^	63.35 ± 7.84^a3b3^	2.12 ± 1.17
CD						
Remission (*n* = 13)	5.82 ± 0.58	1.71 ± 0.64	66.01 ± 9.37	8334.78 ± 3535.38^a1^	58.46 ± 7.16	2.46 ± 0.94
Activity (*n* = 21)	5.75 ± 0.97^a2^	1.30 ± 0.43^a3b1^	71.57 ± 5.79^a3b3^	11971.52 ± 2905.06^a3,b3^	64.79 ± 8.87^a3b3^	2.16 ± 1.14
UC						
Remission (*n* = 10)	5.75 ± 1.05	1.47 ± 0.44	65.28 ± 5.63	8348.40 ± 1301.15	58.34 ± 8.17	1.07 ± 0.50
Activity (*n* = 19)	5.18 ± 1.06^a3b2^	1.56 ± 0.41	67.87 ± 7.07^a3b3^	9679.77 ± 2364.47^a3,b3^	61.70 ± 6.06^a3b3^	2.08 ± 1.27

IBD: inflammatory bowel disease; CD: Crohn's disease; UC: ulcerative colitis. ^a^Compared with the control group. ^b^Compared with patients in remission. ^1^*P* < 0.05, ^2^*P* < 0.01, and ^3^*P* < 0.001.

## Data Availability

The data used to support the findings of the study are available from the corresponding author on request.
